# Atrial Fibrillation Global Changes after Pulmonary Vein and Posterior Wall Isolation: A Charge Density Mapping Study

**DOI:** 10.3390/jcm11102948

**Published:** 2022-05-23

**Authors:** Luigi Pannone, Antonio Bisignani, Antonio Sorgente, Anaïs Gauthey, Domenico G. Della Rocca, Cinzia Monaco, Wim Bories, Robbert Ramak, Ingrid Overeinder, Gezim Bala, Alexandre Almorad, Saverio Iacopino, Gaetano Paparella, Erwin Ströker, Juan Sieira, Panagiotis Flamée, Pedro Brugada, Mark La Meir, Gian-Battista Chierchia, Carlo de Asmundis

**Affiliations:** 1 Heart Rhythm Management Centre, Postgraduate Program in Cardiac Electrophysiology and Pacing, Universitair Ziekenhuis Brussel, Vrije Universiteit Brussel, European Reference Networks Guard-Heart, Laarbeeklaan 101, 1090 Brussels, Belgium; lui.pannone@gmail.com (L.P.); abisignani@hotmail.it (A.B.); sorgente.antonio@gmail.com (A.S.); anaisgauthey@gmail.com (A.G.); domenicodellarocca@hotmail.it (D.G.D.R.); cinziamonaco90@gmail.com (C.M.); wim.bories@acutus.com (W.B.); robbert.ramak@uzbrussel.be (R.R.); ingrid.overeinder@uzbrussel.be (I.O.); gezim.bala@gmail.com (G.B.); alexandre.almorad@uzbrussel.be (A.A.); iacopino@iol.it (S.I.); paparelg@gmail.com (G.P.); erwin.stroker@uzbrussel.be (E.S.); juan.sieira@uzbrussel.be (J.S.); pedro@brugada.org (P.B.); gbchier@yahoo.it (G.-B.C.); 2 Anaesthesiology Department, Universitair Ziekenhuis Brussel, Vrije Universiteit Brussel, 1090 Brussels, Belgium; panagiotis.flamee@uzbrussel.be; 3 Cardiac Surgery Department, Universitair Ziekenhuis Brussel, Vrije Universiteit Brussel, 1090 Brussels, Belgium; lameir@yahoo.com

**Keywords:** atrial fibrillation, charge density, pulmonary vein isolation, posterior wall isolation

## Abstract

Background: Non-contact charge density (CD) mapping allows a global visualization of left atrium (LA) activation and of activation patterns during atrial fibrillation (AF). The aim of this study was to analyze, with CD mapping, the changes in persistent AF induced by pulmonary vein isolation (PVI) and LA posterior wall isolation (LAPWI). Methods: Patients undergoing PVI + LAPWI using the Arctic Front Advance PRO^TM^ cryoballoon system were included in the study. CD maps were created during AF at baseline, after PVI and after LAPWI. Three distinct activation patterns were identified in the CD maps: localized irregular activation (LIA), localized rotational activation (LRA) and focal centrifugal activation (FCA). LA maps were divided into the following regions: anterior, septal, lateral, roof, posterior, inferior. Results: Eleven patients were included, with a total of 33 maps and 198 AF regions analyzed. Global and regional AF cycle lengths significantly increased after PVI and LAPWI. Baseline analysis demonstrated higher LIA, LRA and FCA numbers in the posterior and anterior regions. After PVI, there was no change in LIA, LRA and FCA occurrence. After PVI + LAPWI, a significant decrease in LRA was observed with no difference in LIA and FCA occurrence. In the regional analysis, there was a significant reduction in the LIA number in the inferior region, in the LRA number in the roof and posterior regions and in the FCA number in the lateral region. Conclusions: A global reduction in the LRA number was observed only after PVI + LAPWI; it was driven by a reduction in rotational activity in the roof and posterior regions.

## 1. Introduction

Pulmonary vein isolation (PVI) is the cornerstone of catheter ablation for paroxysmal atrial fibrillation (AF) in the current guidelines [1]; however, PVI has shown suboptimal results in the setting of persistent AF, and this has led to efforts in identifying non-pulmonary vein (PV) triggers capable of sustaining the arrhythmia [2]. 

To improve success rates, left atrial (LA) substrate modification is frequently performed in addition to pulmonary vein isolation (PVI) [2,3]. Furthermore, techniques have been recently developed that seek to detect the underlying mechanism of AF focusing on the identification of specific triggers/electrical factors outside PV [4,5,6].

In this setting, non-contact charge density (CD) mapping (Acutus Medical, Carlsbad, CA, USA) allows a global visualization of the whole chamber activation and analysis of wavefront patterns during AF. This could contribute to designing a more effective, personalized treatment strategy, or to guiding ablation [7,8].

Left atrial posterior wall isolation (LAPWI) in addition to PVI is a feasible strategy that may be considered in the setting of persistent AF. Indeed, structural and electrical abnormalities in the left atrial posterior wall have been reported [9,10,11], namely, slow conduction, a short action potential duration and non-uniform anisotropy [12]. Meta-analyses of randomized and observational studies suggested a benefit of LAPWI compared to PVI alone in persistent AF [12]. However, the results of the individual studies, in particular with radiofrequency, are mixed [12,13,14]. PVI + LAPWI with cryoballoon ablation has demonstrated consistent clinical outcomes, especially in randomized controlled trials [15]. There are several potential advantages to cryoablation [16]. First, lesions created using the cryoballoon are often larger. For this reason, cryoballoon ablation offers a simple technique to ablate and debulk the LAPW. Moreover, cryoenergy can be considered safer with regard to anatomical structures that are close to the LAPW (i.e., the esophagus) [16]. 

The aim of this study was to analyze the mechanism of persistent AF with CD mapping and to characterize the changes in global AF mapping induced by PVI and LAPWI.

## 2. Material and Methods

### 2.1. Study Population

All consecutive patients who underwent second-generation cryoballoon (CB) PVI + LAPWI for persistent AF at Universitair Ziekenhuis Brussel (UZB), between May 2020 and November 2021, were enrolled; they were included in the analysis if the following inclusion criteria were fulfilled: (1) persistent AF defined, following current guidelines, as AF lasting longer than 7 days, including episodes that were terminated by cardioversion, either with drugs or by direct current cardioversion, after 7 days or more [1]; (2) PVI + LAPWI performed with second-generation CB; (3) CD mapping during AF performed with an AcQMap catheter (Acutus Medical, Carlsbad, CA, USA) at baseline, after PVI and after LAPWI.

The exclusion criteria were the following: (1) previous AF ablation or cardiac surgery; (2) paroxysmal or permanent AF; (3) congenital heart disease; (4) non-treated coronary artery disease, by means of a coronary CT scan or coronary angiography; (5) intracavitary thrombus; (6) severe valvular disease, following standard echocardiographic assessment [1,17]; (7) contraindications to general anesthesia, CB or CD mapping. 

This study complied with the Declaration of Helsinki as revised in 2013; the ethic committee approved the study. All patients signed an informed consent. This study was approved by the UZB institutional review board (number 143201524999).

### 2.2. Procedure

All patients underwent PVI + LAPWI using the Arctic Front Advance PRO^TM^ CB system (Medtronic, Inc., Minneapolis, MN, USA). Non-contact CD mapping (AcQMap catheter, Acutus Medical, Carlsbad, CA, USA) was performed at baseline, after PVI and after LAPWI during AF.

### 2.3. Pulmonary Vein Isolation and Left Atrial Posterior Wall Isolation with Cryoballoon

The standard pre-procedural management and ablation with CB have been previously described in detail [18,19,20]. With the exception of amiodarone, antiarrhythmic drugs were stopped a minimum of 5 days before the procedure. All procedures were performed under general anesthesia. A standard protocol was used, as previously described [21]: (1) induction with propofol 1–2 mg/kg titrated to effect (usual dose of 100–200 mg) and (2) propofol infusion 0.02–0.06 mg/kg/min titrated to effect (usual rate of 100–150 cc/h). Following a single transseptal puncture, a 28 mm CB (Arctic Front Advance PRO, Medtronic Inc., Minneapolis, MN, USA) was inserted over an inner lumen mapping catheter (Achieve, Medtronic) in the LA. The CB was advanced, inflated and positioned at each PV ostium. Optimal vessel occlusion was defined by selective contrast injection. Once vessel occlusion was deemed satisfactory, delivery of cryoenergy was started. Standard cryoenergy applications lasted 180 s with a target temperature of −40 °C within the first 60 s. If the temperature did not attain this value, an extra freeze was delivered. In order to avoid phrenic nerve palsy (PNP), diaphragmatic stimulation was achieved by pacing the phrenic nerve during septal PV ablation.

In order to achieve LAPWI, the Achieve mapping catheter was placed deeply in the PVs to stabilize the CB. As per our standard protocol, cryothermal freezes lasted 120 s. The first cryo applications were performed partially overlapping the left superior PV ostium. By a slight clockwise rotation and progressive “pullback” of the sheath while keeping the CB in contact with the posterior wall, consecutive overlapping freezes were applied along the LAPW. The same maneuver was performed from the right superior PV for the superior portion of the LAPW and from the inferior PVs for the inferior portion. The number of freezes to achieve the LAPWI was ≈10–13. At the end of the procedure, after the last CD mapping and subsequent electrical cardioversion, in order to evaluate effective PVI + LAPWI, pacing maneuvers to test entrance and exit block were performed. No additional lesions were needed. During the entire procedure, the activated clotting time was maintained over 350 s by supplementing the heparin infusion as required. Oral anticoagulation was started the same evening of ablation (apixaban in 4 patients, edoxaban in 3 patients, rivaroxaban in 2 patients and dabigatran in 2 patients; all drugs were dosed according to current recommendations [1], and no patients received warfarin).

### 2.4. Non-Contact Charge Density Mapping 

Through a single transseptal puncture, the AcQMap catheter was advanced over a guide wire into the LA via an Acquire (Acutus Medical, Carlsbad, CA, USA) sheath [22,23]. Ultrasound was used to reconstruct the 3D high-resolution atrial anatomy, and the wavefront models were examined in real time with the use of an integral application (AcQTrack Acutus Medical). Global simultaneous recording of atrial activation was performed during continuous ongoing AF at baseline, after PVI and after LAPWI. No AF induction was allowed, and external electrical cardioversion was possible only at the end of the procedure (after LAPWI and last CD mapping). For the current study, LA maps were divided into the following 6 regions: anterior, septal, lateral, roof, posterior, inferior (Supplementary Figure S1). The analysis was performed accordingly, for the global LA map and for each regional AF segment. Five-second AF segments were analyzed. At least three consecutive maps for each segment were acquired to ensure consistency. Three distinct activation patterns were identified and classified by automatic analysis as previously described [4,24]. (1) Localized irregular activation (LIA): a disorganized activation displaying repetitive, multidirectional entry, exit and pivoting conduction through and around a confined (isthmus-like) zone; LIA is met when a difference between the incoming and outgoing wavefront directions is calculated to be more than 90 degrees. (2) Localized rotational activation (LRA): a regionally organized pattern of conduction that rotates, spiraling >270 degrees around a confined central zone. (3) Focal centrifugal activation (FCA): radial activation spreading from a core/focus, which is activated at least 3 ms before its surrounding vertices. In addition, the system-calculated global and regional AF cycle lengths (AFCLs), chamber coherence and recurrence were analyzed.

### 2.5. Statistical Analysis

All variables were tested for normality with the Shapiro–Wilk test. Normally distributed variables were described as the mean ± standard deviation, and the groups were compared through ANOVA or paired or unpaired t-tests as appropriate, while the non-normally distributed variables were described as the median (interquartile range) and compared with the Kruskal–Wallis test, Mann–Whitney test or Wilcoxon signed-rank test as appropriate. The categorical variables were described as frequencies (percentages) and compared with the chi-squared test or Fisher’s exact test as appropriate. 

A *p*-value less than 0.05 was considered statistically significant.

The analysis was performed using R software version 3.6.2 (R Foundation for Statistical Computing, Vienna, Austria).

## 3. Results

### 3.1. Study Population Characteristics

Eleven consecutive patients met the inclusion criteria and were enrolled. All patients presented with AF at baseline ECG. PVI was performed in all patients, and no patient had spontaneous restoration of the sinus rhythm (SR). After PVI, all patients underwent LAPWI, in the same session, with AF persistence in all followed by successful electrical cardioversion to the SR. The complete patient characteristics are summarized in Table 1.

### 3.2. Pulmonary Vein Isolation and Posterior Wall Isolation in Charge Density Map Analysis

**Figure 1:**
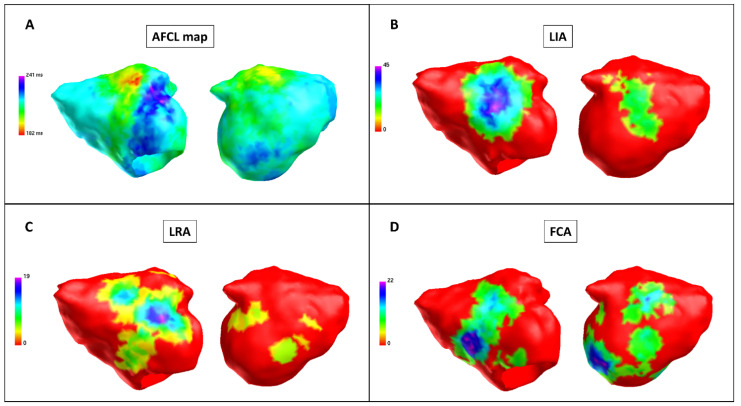
Baseline charge density map (Patient 9). Charge density map with theAcQMap catheter. Each color point on the map corresponds to the color-matched legend on the left. (**A**) Atrial fibrillation cycle length (AFCL) map: left side, anterior-posterior view; right side, posterior-anterior view. An area of a short AFCL in the anterior wall (in red). The AFCL is higher in the posterior and roof regions. Color bar describes AFCL in ms. (**B**) LIA map: left side, anterior-posterior view; right side, posterior-anterior view. Higher LIA number in the anterior and posterior regions. Color bar describes LIA in LIA/s. (**C**) LRA map: left side, anterior-posterior view; right side, posterior-anterior view. Higher LRA number in the anterior and posterior regions. Color bar describes LRA in LRA/s. (**D**) FCA map: left side, anterior-posterior view; right side, posterior-anterior view. Higher FCA number in the anterior and posterior regions. Color bar describes FCA in FCA/s. FCA: focal centrifugal activation; LIA: localized irregular activation; LRA: localized rotational activation.

All patients underwent charge density (CD) mapping at baseline (N = 11), after PVI (N = 11) and after LAPWI (N = 11). All measurements were performed for each of the 6 regions, with a total of 66 regions for each procedural step and 198 AF segments analyzed (Figure 1). 

**Figure 2:**
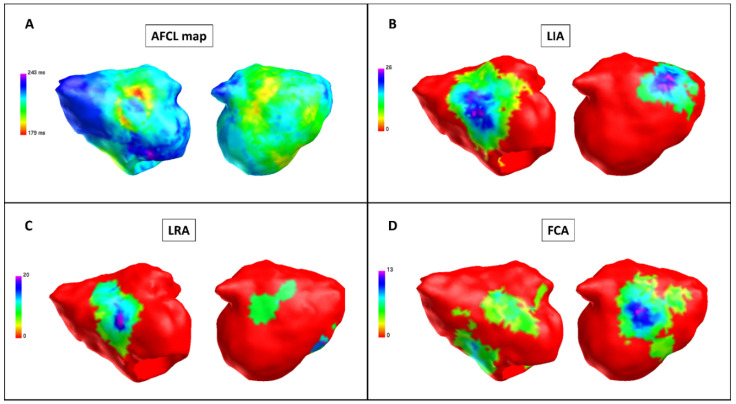
Charge density map after pulmonary vein isolation (Patient 9). Charge density map with the AcQmap^TM^ catheter. Each color point on the map corresponds to the color-matched legend on the left. (**A**) Atrial fibrillation cycle length (AFCL) map: left side, anterior-posterior view; right side, posterior-anterior view. When compared with baseline, the AFCL is higher in the posterior region; there is no area of a short AFCL in the anterior region. Color bar describes AFCL in ms. (**B**) LIA map: left side, anterior-posterior view; right side, posterior-anterior view. Higher LIA number in the anterior and posterior regions. Color bar describes LIA in LIA/s. (**C**) LRA map: left side, anterior-posterior view; right side, posterior-anterior view. Higher LRA number in the anterior and posterior regions. Color bar describes LRA in LRA/s. (**D**) FCA map: left side, anterior-posterior view; right side, posterior-anterior view. Higher FCA number in the anterior and posterior regions. Color bar describes FCA in FCA/s. FCA: focal centrifugal activation; LIA: localized irregular activation; LRA: localized rotational activation.

After PVI, a significant increase was observed in the following measures compared with baseline: global AFCL (198.0 ms ± 17.3 vs. 184.4 ms ± 14.4, *p* < 0.001), regional AFCL (198.0 ms ± 18.7 vs. 184.5 ms ± 14.8, *p* < 0.001). There was no difference in LIA, LRA and FCA occurrence (*p* = 0.77, *p* = 0.58 and *p* = 0.67, respectively) (Figure 2, Table 2).

**Figure 3:**
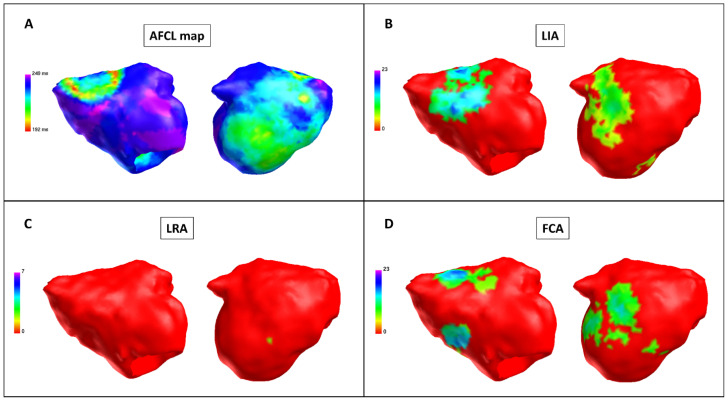
Charge density map after posterior wall isolation (Patient 9). Charge density map with the AcQmap^TM^ catheter after pulmonary vein isolation (PVI) + left atrial posterior wall isolation (LAPWI). Each color point on the map corresponds to the color-matched legend on the left. (**A**) Atrial fibrillation cycle length (AFCL) map: left side, anterior-posterior view; right side, posterior-anterior view. When compared with the baseline and post-PVI maps, the AFCL is higher, especially in the roof and posterior regions. Color bar describes AFCL in ms. (**B**) LIA map: left side, anterior-posterior view; right side, posterior-anterior view. After PVI + LAPWI, there is a significant reduction in LIA in the inferior region. Color bar describes LIA in LIA/s. (**C**) LRA map: left side, anterior-posterior view; right side, posterior-anterior view. After PVI + LAPWI, there is a significant reduction in LRA in the inferior region. Color bar describes LRA in LRA/s. (**D**) FCA map: left side, anterior-posterior view; right side, posterior-anterior view. Higher FCA number in the anterior and posterior regions with no significant change after PVI + LAPWI. Color bar describes FCA in FCA/s. FCA: focal centrifugal activation; LIA: localized irregular activation; LRA: localized rotational activation.

After PVI + LAPWI, a significant increase was observed in the following measures compared with baseline: global AFCL (201.6 ms ± 17.3 vs. 184.4 ms ± 14.4, *p* < 0.001), regional AFCL (201.8 ms ± 17.8 vs. 184.5 ms ± 14.8, *p* < 0.001). Furthermore, compared with baseline, a significant decrease in LRA was observed after PVI + LAPWI (6.2 LRA ± 6.2 vs. 9.8 LRA ± 9.8, *p* = 0.04); there was no difference in LIA and FCA occurrence (*p* = 0.48 and *p* = 0.72, respectively) (Figure 3, Table 3).

### 3.3. Regional Charge Density Map Analysis

Baseline regional map analysis demonstrated a higher LIA number in the posterior and anterior regions (74.7 LIA ± 33.2 and 61.9 LIA ± 31.8, respectively, *p* < 0.001), a higher LRA number in the posterior and anterior regions (16.9 LRA ± 9.8 and 16.0 LRA ± 12.6, respectively, *p* < 0.001) and a higher FCA number in the posterior and anterior regions (23.3 FCA ± 8.7 and 29.0 FCA ± 13.3, respectively, *p* < 0.001) (Supplementary Table S1). 

Map analysis after PVI demonstrated a higher LIA number in the posterior and anterior regions (65.2 LIA ± 40.3 and 76.6 LIA ± 44.4, respectively, *p* < 0.001), a higher LRA number in the posterior and anterior regions (12.6 LRA ± 14.5 and 17.3 LRA ± 17.4, respectively, *p* = 0.031) and a higher FCA number in the posterior and anterior regions (26.1 FCA ± 11.8 and 25.6 FCA ± 13.0, respectively, *p* < 0.001) (Supplementary Table S1). Compared with baseline, after PVI, there was no regional change in LIA, LRA and FCA occurrence (Supplementary Table S2).

**Figure 4:**
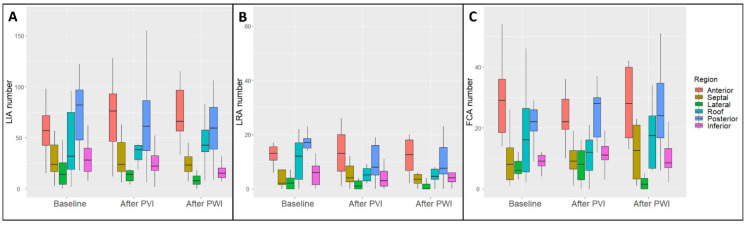
Regional analysis of abnormal activation patterns in charge density mapping. Each color box in the graph corresponds to the color-matched region (legend on the right). (**A**) LIA absolute number by region and procedural step, namely, baseline, after pulmonary vein isolation (PVI) and after left atrial posterior wall isolation (LAPWI). (**B**) LRA absolute number by region and procedural step, namely, baseline, after PVI and after LAPWI. (**C**) FCA absolute number by region and procedural step, namely, baseline, after pulmonary vein isolation (PVI) and after LAPWI. FCA: focal centrifugal activation; LIA: localized irregular activation; LRA: localized rotational activation. The midline of each boxplot is the median, with the upper and lower limits of the box being the 3rd and 1st quartiles (75th and 25th percentiles), respectively. By default, the whiskers will extend up to 1.5 times the interquartile range from the top (bottom) of the box to the furthest datum within that distance.

Map analysis after LAPWI demonstrated a higher LIA number in the posterior and anterior regions (59.6 LIA ± 32.2 and 72.6 LIA ± 28.6, respectively, *p* < 0.001), a higher LRA number in the posterior and anterior regions (10.1 LRA ± 7.9 and 11.8 LRA ± 7.0, respectively, *p* = 0.002) and a higher FCA number in the posterior and anterior regions (26.6 FCA ± 15.2 and 27.8 FCA ± 12.3, respectively, *p* < 0.001) (Supplementary Table S1). Compared with baseline, after PVI + LAPWI, there was a significant reduction in the LIA number in the inferior region (16.2 LIA ± 8.1 vs. 32.7 LIA ± 22.1, respectively, *p* = 0.011), in the LRA number in the roof region (5.5 LRA ± 4.3 vs. 11.0 LRA ± 8.4, respectively, *p* = 0.03) and in the posterior region (10.1 LRA ± 7.9 vs. 16.9 LRA ± 9.8, respectively, *p* = 0.042), and in the FCA number in the lateral region (2.6 FCA ± 3.5 vs. 7.3 FCA ± 3.1, respectively, *p* = 0.027) (Figure 4 and Supplementary Table S2).

## 4. Discussion

The current study is the first to analyze the global changes in AF after PVI and LAPWI with CD mapping. The main findings of this study are summarized as follows: (1) after PVI and LAPWI, a significant increase in the AFCL was observed; (2) there was no difference in LIA, LRA and FCA occurrence after PVI; (3) a global reduction in the LRA number was observed only after PVI + LAPWI, and it was significant in the roof and posterior regions.

### 4.1. Global Charge Density Mapping in Persistent Atrial Fibrillation 

Pulmonary vein triggers have been demonstrated as the main mechanism of paroxysmal AF, and thus PVI is the first-line approach for AF catheter ablation [25]. However, a PVI-only strategy has shown suboptimal results in patients with persistent AF, and the time between the first AF episode and ablation is a known predictor of recurrence [26]. This might be explained by the different mechanism of persistent AF that often goes beyond the pulmonary veins. In particular, electrical remodeling with shortening of the atrial effective refractory period and dispersion of refractoriness has been demonstrated to self-perpetuate AF [27]. Furthermore, shortening of the action potential duration and Ca^2+^-handling abnormalities have been demonstrated in persistent AF. Finally, structural remodeling and slow conduction contribute to the increase in the stability of reentrant waves and to AF perpetuation [28,29,30]. Consistent with these findings, studies with organ-level atrial modeling from cardiac computed tomography scans of AF patients, identified PVI with LAPWI as an effective strategy [31]. Based on in silico predictive models, the optimal ablation approach resulting in termination or, if that is not possible, in atrial tachycardia conversion was as follows: 20% PVI alone, 6% box ablation and 46% all driver hotspots [32].

Furthermore, the role of non-pulmonary vein triggers, located in the LAPW, has been demonstrated to contribute to the pathogenesis of persistent AF [33].

CD mapping provides a global simultaneous map of in vivo human AF. Previous studies with CD mapping in persistent AF demonstrated that LIA was found in all LA regions with a higher number on the anterior and posterior walls [24]. 

In the current study, higher LIA, LRA and FCA numbers were observed in the anterior and posterior regions at baseline, after PVI and after LAPWI. This is consistent with previous findings [24] and points towards the anterior and posterior walls as sites harboring the substrate for persistent AF maintenance.

Furthermore, a prolongation in the AFCL was observed after PVI and LAPWI. A higher AFCL has been associated with termination to sinus rhythm during ablation in persistent AF, and it is a predictor of long-term ablation success [34]. 

### 4.2. The Role of Pulmonary Vein Isolation and Posterior Wall Isolation in Persistent Atrial Fibrillation

Although the recurrence rate after PVI is higher in persistent AF, it is the first recommended procedural step [1]. However, a PVI-only approach does not target extrapulmonary vein substrates; accordingly, LRA, LIA and FCA were not affected by PVI in the current study, both in the global and regional analyses. 

The optimal ablation strategy in non-paroxysmal AF remains controversial; the STAR AF II trial demonstrated that in persistent AF, ablation of electrograms showing complex fractionated activity or linear ablation across the left atrial roof and mitral valve isthmus as an add-on to PVI did not improve the outcome as compared to PVI only [2]. This result can be explained by the complex and variable nature of non-PV triggers, which cannot be targeted by empirical ablation but require a personalized, patient-specific approach. 

Recently, the MARSHALL-PLAN study [3], with a standard set of radiofrequency lesions (vein of Marshall, coronary sinus, roof, mitral and cavotricuspid isthmus) reported a success rate of 79% after 1 year; this may be related to the extensive atrial lesions including the anterior wall and LAPW, that have been shown by the current study as the main sites of abnormal activities.

Finally, our group demonstrated that a single procedure of PVI + LAPWI with a cryoballoon is safe and effective for persistent AF [18,20]. The rationale might be found in the reduction in the global LRA number that was observed only after PVI + LAPWI in the current study; furthermore, after PVI + PWI, there was a significant reduction in the LIA number in the inferior region, in the LRA number in the roof and posterior regions and in the FCA number in the lateral region. Rotational activity and posterior wall triggers are known substrates in persistent AF [28,33] that can be targeted successfully with a cryoballoon PVI + LAPWI single-procedure strategy; this might lead to improved clinical outcomes.

## 5. Limitations

The number of patients included was relatively small. However, the analysis was performed on a relatively high number of AF segments. Clinical follow-up was not reported, being beyond the scope of this paper, but clinical studies with PVI and LAPWI guided by CD mapping data are eagerly awaited.

## 6. Conclusions

At CD mapping analysis, a significant increase in the AFCL was observed after PVI and LAPWI. LIA, LRA and FCA were not affected by PVI. A global reduction in the LRA number was observed only after PVI + LAPWI; it was driven by a substrate modification in the roof and posterior regions. Further studies are eagerly awaited to evaluate the clinical outcome after PVI + LAPWI guided by CD mapping. Future research should aim at a personalized, patient-tailored approach in AF ablation. In particular, a prospective randomized clinical trial might be theoretically designed to compare the PVI-only vs. the LAPWI-only approach.

## Figures and Tables

**Table 1:** Clinical characteristics of patients.

	Overall (N = 11)
Age (years)	59.2 ± 9.9
Gender (male)	9 (81.8%)
Height (cm)	183.0 ± 3.9
Weight (Kg)	95.2 ± 12.7
BMI	29.4 ± 3.9
CHA_2_DS_2_VASc	1.8 ± 0.8
Hypertension	9 (81.8%)
Diabetes	3 (27.3%)
Dyslipidemia	6 (54.5%)
Heart failure	1 (9.1%)
Chronic kidney disease	2 (18.2%)
Coronary artery disease	1 (9.1%)
Valvular heart disease	0 (0.0%)
COPD	1 (9.1%)
TIA or CVA	0 (0.0%)
Drugs	
AAD Class Ic	2 (18.2%)
Betablockers	6 (54.5%)
AAD Class III	4 (36.4%)
AAD Class IV	0 (0.0%)
OAC	11 (100.0%)
VKA	0 (0.0%)
DOACs	11 (100.0%)
Aspirin	1 (9.1%)
ACEI or ARBs	8 (2.7%)
LVEF (%)	51.0 ± 16.4
LA volume index (mL/mq)	43.8 ± 12.0

AAD: antiarrhythmic drugs; BMI: body mass index; COPD: chronic obstructive pulmonary disease; CVA: cerebrovascular accident; DOACs: direct oral anticoagulants; LA: left atrium; LVEF: left ventricular ejection fraction; OAC: oral anticoagulation; TIA: transient ischemic attack; VKA: vitamin K antagonists.

**Table 2:** Charge density map data pre- and post-pulmonary vein isolation.

	Baseline (N = 66)	Post-PVI (N = 66)	Baseline + Post-PVI (N = 132)	*p* Value
Global AFCL mean (ms)	184.4 ± 14.4	198.0 ± 17.3	191.2 ± 17.2	<0.001 *
Regional AFCL mean (ms)	184.5 ± 14.8	198.0 ± 18.7	191.2 ± 18.1	<0.001 *
Global intra-chamber coherence mean	0.4 ± 0.1	0.4 ± 0.1	0.4 ± 0.1	0.073
Regional intra-chamber coherence mean	0.4 ± 0.1	0.4 ± 0.2	0.4 ± 0.1	0.095
Global recurrence mean	0.4 ± 0.1	0.4 ± 0.1	0.4 ± 0.1	0.078
Regional recurrence mean	0.6 ± 0.1	0.6 ± 0.1	0.6 ± 0.1	0.078
LIA number (N)	43.5 ± 32.4	42.9 ± 35.4	43.2 ± 33.8	0.92
LRA number (N)	9.8 ± 9.8	8.7 ± 11.4	9.3 ± 10.6	0.53
Focal number (N)	16.3 ± 12.6	15.6 ± 11.5	15.9 ± 12.1	0.74
LIA perc time (N)	12.5 ± 10.0	11.9 ± 10.5	12.2 ± 10.2	0.77
LRA perc time (N)	5.4 ± 5.7	4.8 ± 6.3	5.1 ± 6.0	0.58
Focal perc time (N)	2.0 ± 1.6	1.9 ± 1.4	2.0 ± 1.5	0.67

AFCL: atrial fibrillation cycle length; FCA: focal centrifugal activation; LIA: localized irregular activation; LRA: localized rotational activation; PVI: pulmonary vein isolation. * significant *p* Value.

**Table 3:** Charge density map data pre- and post-posterior wall isolation.

	Baseline (N = 66)	Post-PVI + LAPWI (N = 66)	Baseline + Post-PVI + LAPWI (N = 132)	*p* Value
Global AFCL mean (ms)	184.4 ± 14.4	201.6 ± 16.8	192.1 ± 18.0	<0.001 *
Regional AFCL mean (ms)	184.5 ± 14.8	201.8 ± 17.8	192.2 ± 18.6	<0.001 *
Global intra-chamber coherence mean	0.4 ± 0.1	0.4 ± 0.1	0.4 ± 0.1	0.089
Regional intra-chamber coherence mean	0.4 ± 0.1	0.4 ± 0.1	0.4 ± 0.1	0.096
Global recurrence mean	0.4 ± 0.1	0.4 ± 0.1	0.4 ± 0.1	0.15
Regional recurrence mean	0.6 ± 0.1	0.6 ± 0.1	0.6 ± 0.1	0.25
LIA number (N)	43.5 ± 32.4	37.8 ± 30.7	40.2 ± 31.3	0.48
LRA number (N)	9.8 ± 9.8	6.2 ± 6.2	8.0 ± 8.4	0.04 *
Focal number (N)	16.3 ± 12.6	16.2 ± 13.4	16.7 ± 13.0	0.72
LIA perc time (N)	12.5 ± 10.0	10.6 ± 8.8	11.5 ± 9.4	0.41
LRA perc time (N)	5.4 ± 5.7	3.5 ± 3.9	4.4 ± 5.0	0.094
Focal perc time (N)	2.0 ± 1.6	2.0 ± 1.6	2.1 ± 1.6	0.75

AFCL: atrial fibrillation cycle length; FCA: focal centrifugal activation; LAPWI: left atrial posterior wall isolation; LIA: localized irregular activation; LRA: localized rotational activation; PVI: pulmonary vein isolation. * significant *p* Value.

## Data Availability

Data are contained within the article or Supplementary Materials.

## Supplementary Materials

The following supporting information can be downloaded at: https://www.mdpi.com/article/10.3390/jcm11102948/s1, Figure S1: Left atrium regional segmentation; Table S1: Charge density map data by region and procedural step; Table S2: Charge density map data by procedural step and region.
