# Supplementary material for: Atrial Fibrillation Global Changes after Pulmonary Vein and Posterior Wall Isolation: A Charge Density Mapping Study
Source: J Clin Med. 2022 May 23;11(10):2948. doi: 10.3390/jcm11102948 (PMC9145946; doi:10.3390/jcm11102948)
Supplement: Supplementary file 1 [file jcm-11-02948-s001.zip › jcm-1698959-supplementary.pdf]

SUPPLEMENTARY TABLES

Supplementary Table S1.

Charge density map data by region and procedural step

Procedural step		Anterior (N=33)	Septal (N=33)	Lateral (N=33)	Roof (N=33)	Posterior (N=33)	Inferior (N=33)	Total (N=198)	p value
Baseline	Global AFCL mean (ms)	184.4 ± 15.0	184.4 ± 15.0	184.4 ± 15.0	184.4 ± 15.0	184.4 ± 15.0	184.4 ± 15.0	184.4 ± 14.4	1.00
	Global AFCL SD (ms)	6.5 ± 1.9	6.5 ± 1.9	6.5 ± 1.9	6.5 ± 1.9	6.5 ± 1.9	6.5 ± 1.9	6.5 ± 1.8	1.00
	Global AFCL CovC	3.6 ± 1.1	3.6 ± 1.1	3.6 ± 1.1	3.6 ± 1.1	3.6 ± 1.1	3.6 ± 1.1	3.6 ± 1.1	1.00
	Regional AFCL mean (ms)	185.4 ± 15.5	184.9 ± 16.9	188.1 ± 14.6	180.1 ± 14.8	182.1 ± 14.5	186.4 ± 14.6	184.5 ± 14.8	0.84
	Regional AFCL SD (ms)	6.6 ± 2.6	4.7 ± 2.2	4.7 ± 1.8	4.0 ± 1.9	5.3 ± 2.5	5.4 ± 2.3	5.1 ± 2.3	0.16
	Regional AFCL CovC	3.5 ± 1.3	2.5 ± 1.3	2.5 ± 1.0	2.2 ± 0.9	2.9 ± 1.5	3.0 ± 1.4	2.8 ± 1.3	0.20
	Global Intra Chamber Coherence mean	0.4 ± 0.1	0.4 ± 0.1	0.4 ± 0.1	0.4 ± 0.1	0.4 ± 0.1	0.4 ± 0.1	0.4 ± 0.1	1.00

**Global Intra Chamber Coherence max**	0.5 ± 0.1	0.5 ± 0.1	0.5 ± 0.1	0.5 ± 0.1	0.5 ± 0.1	0.5 ± 0.1	0.5 ± 0.1	1.00
**Global Intra Chamber Coherence SD**	0.1 ± 0.0	0.1 ± 0.0	0.1 ± 0.0	0.1 ± 0.0	0.1 ± 0.0	0.1 ± 0.0	0.1 ± 0.0	1.00
**Regional Intra Chamber Coherence mean**	0.3 ± 0.1	0.4 ± 0.1	0.4 ± 0.1	0.4 ± 0.1	0.4 ± 0.1	0.4 ± 0.1	0.4 ± 0.1	0.93
**Regional Intra Chamber Coherence max**	0.5 ± 0.1	0.5 ± 0.1	0.5 ± 0.1	0.5 ± 0.1	0.5 ± 0.1	0.5 ± 0.1	0.5 ± 0.1	0.99
**Regional Intra Chamber Coherence SD**	0.1 ± 0.0	0.1 ± 0.0	0.0 ± 0.0	0.1 ± 0.0	0.1 ± 0.0	0.0 ± 0.0	0.1 ± 0.0	0.56
**Global Recurrence Mean**	0.4 ± 0.1	0.4 ± 0.1	0.4 ± 0.1	0.4 ± 0.1	0.4 ± 0.1	0.4 ± 0.1	0.4 ± 0.1	1.00
**Global Recurrence SD**	0.2 ± 0.0	0.2 ± 0.0	0.2 ± 0.0	0.2 ± 0.0	0.2 ± 0.0	0.2 ± 0.0	0.2 ± 0.0	1.00
**Regional Recurrence Mean**	0.5 ± 0.1	0.6 ± 0.1	0.7 ± 0.1	0.6 ± 0.1	0.5 ± 0.1	0.7 ± 0.1	0.6 ± 0.1	< 0.001
**Regional Recurrence SD**	0.2 ± 0.0	0.2 ± 0.0	0.2 ± 0.0	0.2 ± 0.0	0.2 ± 0.0	0.2 ± 0.0	0.2 ± 0.0	0.09
**LIA occ number (cutoff 0) (N)**	61.9 ± 31.8	28.6 ± 17.5	17.1 ± 15.4	45.8 ± 32.8	74.7 ± 33.2	32.7 ± 22.1	43.5 ± 32.4	< 0.001
**LRA occ number (cutoff 0) (N)**	16.0 ± 12.6	6.9 ± 9.1	2.4 ± 2.6	11.0 ± 8.4	16.9 ± 9.8	5.9 ± 4.5	9.8 ± 9.8	< 0.001
**Focal occ number (cutoff 0) (N)**	29.0 ± 13.3	9.6 ± 7.7	7.3 ± 3.1	17.7 ± 15.6	23.3 ± 8.7	10.9 ± 8.2	16.3 ± 12.6	< 0.001

**LIA perc time (cutoff 0) (N)**	17.9 ± 10.8	8.1 ± 5.6	4.6 ± 4.2	13.2 ± 9.7	21.7 ± 10.8	9.1 ± 6.5	12.5 ± 10.0	< 0.001
**LRA perc time (cutoff 0) (N)**	9.3 ± 8.1	3.8 ± 5.4	1.1 ± 1.4	6.0 ± 4.6	8.7 ± 5.5	3.2 ± 2.7	5.4 ± 5.7	0.001
**Focal perc time (cutoff 0) (N)**	3.6 ± 1.7	1.2 ± 0.9	0.9 ± 0.4	2.2 ± 1.9	2.9 ± 1.1	1.3 ± 1.0	2.0 ± 1.6	< 0.001
**LIA occ number (cutoff 5) (N)**	56.5 ± 29.4	24.5 ± 17.3	11.5 ± 12.4	43.8 ± 34.2	71.5 ± 34.1	26.4 ± 22.5	39.0 ± 32.6	< 0.001
**LRA occ number (cutoff 5) (N)**	13.5 ± 11.6	5.8 ± 8.7	1.8 ± 2.1	10.1 ± 8.3	15.2 ± 8.7	5.3 ± 4.6	8.6 ± 9.0	0.001
**Focal occ number (cutoff 5) (N)**	26.2 ± 12.8	8.0 ± 7.5	5.3 ± 3.0	17.3 ± 16.0	21.6 ± 9.1	9.0 ± 8.2	14.6 ± 12.5	< 0.001
**LIA perc time (cutoff 5) (N)**	16.7 ± 10.1	7.1 ± 5.5	3.2 ± 3.5	12.7 ± 10.1	20.9 ± 11.0	7.6 ± 6.7	11.4 ± 10.0	< 0.001
**LRA perc time (cutoff 5) (N)**	8.1 ± 7.6	3.3 ± 5.3	0.9 ± 1.1	5.5 ± 4.5	8.2 ± 5.2	2.9 ± 2.8	4.8 ± 5.4	0.003
**Focal perc time (cutoff 5) (N)**	3.2 ± 1.6	1.0 ± 0.9	0.6 ± 0.4	2.1 ± 2.0	2.7 ± 1.1	1.1 ± 1.0	1.8 ± 1.6	< 0.001
**LIA occ number (cutoff 30) (N)**	31.0 ± 30.0	6.7 ± 9.8	4.7 ± 9.1	28.5 ± 27.7	54.5 ± 28.5	14.6 ± 18.8	23.3 ± 27.6	< 0.001

	**LRA occ number (cutoff 30) (N)**	10.4 ± 10.5	3.1 ± 6.2	0.3 ± 0.9	6.5 ± 6.6	10.5 ± 6.8	2.4 ± 2.8	5.5 ± 7.3	< 0.001
	**Focal occ number (cutoff 30) (N)**	20.6 ± 12.3	4.6 ± 5.3	1.9 ± 2.5	14.2 ± 15.2	15.6 ± 10.2	3.5 ± 3.9	10.1 ± 11.5	< 0.001
	**LIA perc time (cutoff 30) (N)**	9.8 ± 10.2	2.0 ± 3.1	1.3 ± 2.6	8.7 ± 8.7	16.6 ± 9.5	4.4 ± 5.8	7.1 ± 8.8	< 0.001
	**LRA perc time (cutoff 30) (N)**	6.4 ± 7.2	1.9 ± 3.9	0.1 ± 0.4	3.6 ± 3.7	5.7 ± 4.1	1.4 ± 1.8	3.2 ± 4.6	0.003
	**Focal perc time (cutoff 30) (N)**	2.6 ± 1.5	0.6 ± 0.7	0.2 ± 0.3	1.8 ± 1.9	1.9 ± 1.3	0.4 ± 0.5	1.3 ± 1.4	< 0.001
**Post PVI**	**Global AFCL mean (ms)**	198.0 ± 18.0	198.0 ± 18.0	198.0 ± 18.0	198.0 ± 18.0	198.0 ± 18.0	198.0 ± 18.0	198.0 ± 17.3	1.00
	**Global AFCL SD (ms)**	9.6 ± 4.4	9.6 ± 4.4	9.6 ± 4.4	9.6 ± 4.4	9.6 ± 4.4	9.6 ± 4.4	9.6 ± 4.3	1.00
	**Global AFCL CovC**	4.8 ± 2.0	4.8 ± 2.0	4.8 ± 2.0	4.8 ± 2.0	4.8 ± 2.0	4.8 ± 2.0	4.8 ± 1.9	1.00
	**Regional AFCL mean (ms)**	197.0 ± 19.5	196.7 ± 18.0	204.7 ± 21.7	194.8 ± 20.2	196.8 ± 18.2	198.0 ± 17.2	198.0 ± 18.7	0.87
	**Regional AFCL SD (ms)**	6.8 ± 2.9	5.0 ± 2.3	7.2 ± 4.2	4.7 ± 3.3	7.6 ± 4.1	5.8 ± 2.3	6.2 ± 3.3	0.20
	**Regional AFCL CovC**	3.4 ± 1.5	2.5 ± 1.0	3.5 ± 2.0	2.4 ± 1.6	3.8 ± 1.9	2.9 ± 1.0	3.1 ± 1.6	0.19

**Global Intra Chamber Coherence mean**	0.4 ± 0.1	0.4 ± 0.1	0.4 ± 0.1	0.4 ± 0.1	0.4 ± 0.1	0.4 ± 0.1	0.4 ± 0.1	1.00
**Global Intra Chamber Coherence max**	0.5 ± 0.1	0.5 ± 0.1	0.5 ± 0.1	0.5 ± 0.1	0.5 ± 0.1	0.5 ± 0.1	0.5 ± 0.1	1.00
**Global Intra Chamber Coherence SD**	0.1 ± 0.0	0.1 ± 0.0	0.1 ± 0.0	0.1 ± 0.0	0.1 ± 0.0	0.1 ± 0.0	0.1 ± 0.0	1.00
**Regional Intra Chamber Coherence mean**	0.4 ± 0.2	0.4 ± 0.2	0.4 ± 0.1	0.4 ± 0.2	0.4 ± 0.2	0.4 ± 0.1	0.4 ± 0.2	0.99
**Regional Intra Chamber Coherence max**	0.5 ± 0.2	0.5 ± 0.2	0.5 ± 0.2	0.5 ± 0.2	0.5 ± 0.1	0.5 ± 0.2	0.5 ± 0.1	0.99
**Regional Intra Chamber Coherence SD**	0.1 ± 0.0	0.0 ± 0.0	0.0 ± 0.0	0.0 ± 0.0	0.1 ± 0.0	0.0 ± 0.0	0.1 ± 0.0	0.19
**Global Recurrence Mean**	0.4 ± 0.1	0.4 ± 0.1	0.4 ± 0.1	0.4 ± 0.1	0.4 ± 0.1	0.4 ± 0.1	0.4 ± 0.1	1.00
**Global Recurrence SD**	0.2 ± 0.0	0.2 ± 0.0	0.2 ± 0.0	0.2 ± 0.0	0.2 ± 0.0	0.2 ± 0.0	0.2 ± 0.0	1.00
**Regional Recurrence Mean**	0.6 ± 0.2	0.7 ± 0.1	0.8 ± 0.1	0.7 ± 0.1	0.6 ± 0.1	0.7 ± 0.1	0.6 ± 0.1	< 0.001
**Regional Recurrence SD**	0.2 ± 0.0	0.2 ± 0.0	0.2 ± 0.0	0.2 ± 0.1	0.2 ± 0.0	0.2 ± 0.0	0.2 ± 0.0	0.88
**LIA occ number (cutoff 0) (N)**	76.6 ± 44.4	35.0 ± 27.1	17.4 ± 14.(	38.4 ± 21.5	65.2 ± 40.3	24.7 ± 14.1	42.9 ± 35.4	< 0.001
**LRA occ number (cutoff 0) (N)**	17.3 ± 17.4	6.4 ± 6.3	3.2 ± 5.4	7.5 ± 8.7	12.6 ± 14.5	5.1 ± 5.5	8.7 ± 11.4	0.03
**Focal occ number (cutoff 0) (N)**	25.6 ± 13.0	11.3 ± 8.7	8.1 ± 5.7	10.9 ± 7.1	26.1 ± 11.8	11.5 ± 6.5	15.6 ± 11.5	< 0.001

**LIA perc time (cutoff 0) (N)**	21.8 ± 14.0	9.1 ± 7.3	4.7 ± 4.1	11.2 ± 6.9	18.2 ± 12.0	6.7 ± 3.9	11.9 ± 10.5	< 0.001
**LRA perc time (cutoff 0) (N)**	9.7 ± 9.6	3.4 ± 3.3	1.6 ± 2.5	4.7 ± 6.6	6.4 ± 6.7	2.9 ± 3.2	4.8 ± 6.3	0.027
**Focal perc time (cutoff 0) (N)**	3.1 ± 1.6	1.4 ± 1.0	0.9 ± 0.6	1.3 ± 0.9	3.2 ± 1.4	1.4 ± 0.8	1.9 ± 1.4	< 0.001
**LIA occ number (cutoff 5) (N)**	71.7 ± 43.9	26.6 ± 21.8	11.7 ± 9.0	34.2 ± 20.4	60.4 ± 38.6	17.5 ± 10.8	37.0 ± 34.4	< 0.001
**LRA occ number (cutoff 5) (N)**	16.3 ± 16.8	4.8 ± 5.4	2.5 ± 4.8	6.7 ± 8.0	11.4 ± 13.5	4.5 ± 5.6	7.7 ± 10.8	0.022
**Focal occ number (cutoff 5) (N)**	22.7 ± 13.7	9.7 ± 8.1	6.7 ± 5.8	9.4 ± 7.0	23.5 ± 12.9	10.5 ± 6.1	13.8 ± 11.4	< 0.001
**LIA perc time (cutoff 5) (N)**	20.8 ± 14.0	7.1 ± 6.0	3.3 ± 2.7	10.3 ± 6.9	17.1 ± 11.8	4.8 ± 2.9	10.6 ± 10.4	< 0.001
**LRA perc time (cutoff 5) (N)**	9.4 ± 9.4	2.6 ± 2.8	1.2 ± 2.1	4.3 ± 6.3	5.9 ± 6.3	2.6 ± 3.2	4.3 ± 6.1	0.017
**Focal perc time (cutoff 5) (N)**	2.8 ± 1.7	1.2 ± 1.0	0.8 ± 0.7	1.2 ± 0.8	2.9 ± 1.5	1.3 ± 0.8	1.7 ± 1.4	< 0.001
**LIA occ number (cutoff 30) (N)**	51.3 ± 38.6	10.4 ± 11.3	3.0 ± 5.2	22.5 ± 22.1	40.2 ± 30.0	6.5 ± 6.4	22.3 ± 28.3	< 0.001
**LRA occ number (cutoff 30) (N)**	11.3 ± 14.4	2.3 ± 3.2	1.4 ± 4.2	4.0 ± 7.8	7.8 ± 8.9	3.0 ± 4.5	5.0 ± 8.6	0.045
**Focal occ number (cutoff 30) (N)**	15.7 ± 14.5	6.5 ± 8.5	3.6 ± 4.5	6.2 ± 6.5	16.3 ± 12.7	6.6 ± 5.5	9.2 ± 10.4	0.007

	**LIA perc time (cutoff 30) (N)**	15.7 ± 12.9	2.8 ± 3.1	0.8 ± 1.4	6.9 ± 7.5	12.1 ± 9.9	1.8 ± 1.8	6.7 ± 9.1	< 0.001
	**LRA perc time (cutoff 30) (N)**	6.7 ± 8.4	1.2 ± 1.8	0.6 ± 1.8	2.8 ± 6.4	4.5 ± 4.8	1.7 ± 2.6	2.9 ± 5.2	0.055
	**Focal perc time (cutoff 30) (N)**	1.9 ± 1.8	0.8 ± 1.0	0.5 ± 0.6	0.8 ± 0.8	2.0 ± 1.5	0.8 ± 0.7	1.1 ± 1.3	0.007
**Post PWI**	**Global AFCL mean (ms)**	201.6 ± 17.8	201.6 ± 17.8	201.6 ± 17.8	201.6 ± 17.8	201.6 ± 17.8	201.6 ± 17.8	201.6 ± 16.8	1.00
	**Global AFCL SD (ms)**	7.9 ± 4.2	7.9 ± 4.2	7.9 ± 4.2	7.9 ± 4.2	7.9 ± 4.2	7.9 ± 4.2	7.9 ± 4.0	1.00
	**Global AFCL CovC**	3.9 ± 2.1	3.9 ± 2.1	3.9 ± 2.1	3.9 ± 2.1	3.9 ± 2.1	3.9 ± 2.1	3.9 ± 1.9	1.00
	**Regional AFCL mean (ms)**	200.1 ± 20.8	200.8 ± 19.3	208.9 ± 20.1	199.0 ± 15.7	200.0 ± 15.3	202.0 ± 18.8	201.8 ± 17.8	0.90
	**Regional AFCL SD (ms)**	6.7 ± 4.0	5.2 ± 3.7	6.0 ± 3.7	6.7 ± 4.8	6.1 ± 2.9	5.9 ± 3.9	6.1 ± 3.7	0.97
	**Regional AFCL CovC**	3.4 ± 2.2	2.6 ± 1.9	2.8 ± 1.7	3.3 ± 2.4	3.1 ± 1.6	2.9 ± 1.7	3.0 ± 1.9	0.97
	**Global Intra Chamber Coherence mean**	0.4 ± 0.1	0.4 ± 0.1	0.4 ± 0.1	0.4 ± 0.1	0.4 ± 0.1	0.4 ± 0.1	0.4 ± 0.1	1.00
	**Global Intra Chamber Coherence max**	0.5 ± 0.1	0.5 ± 0.1	0.5 ± 0.1	0.5 ± 0.1	0.5 ± 0.1	0.5 ± 0.1	0.5 ± 0.1	1.00
	**Global Intra Chamber Coherence SD**	0.1 ± 0.0	0.1 ± 0.0	0.1 ± 0.0	0.1 ± 0.0	0.1 ± 0.0	0.1 ± 0.0	0.1 ± 0.0	1.00

**Regional Intra Chamber Coherence mean**	0.4 ± 0.2	0.4 ± 0.2	0.5 ± 0.1	0.4 ± 0.1	0.4 ± 0.2	0.4 ± 0.1	0.4 ± 0.1	0.87
**Regional Intra Chamber Coherence max**	0.5 ± 0.2	0.5 ± 0.1	0.5 ± 0.1	0.5 ± 0.1	0.5 ± 0.1	0.5 ± 0.1	0.5 ± 0.1	0.99
**Regional Intra Chamber Coherence SD**	0.1 ± 0.0	0.1 ± 0.0	0.0 ± 0.0	0.1 ± 0.0	0.1 ± 0.0	0.0 ± 0.0	0.1 ± 0.0	0.29
**Global Recurrence Mean**	0.4 ± 0.1	0.4 ± 0.1	0.4 ± 0.1	0.4 ± 0.1	0.4 ± 0.1	0.4 ± 0.1	0.4 ± 0.1	1.00
**Global Recurrence SD**	0.2 ± 0.0	0.2 ± 0.0	0.2 ± 0.0	0.2 ± 0.0	0.2 ± 0.0	0.2 ± 0.0	0.2 ± 0.0	1.00
**Regional Recurrence Mean**	0.6 ± 0.1	0.7 ± 0.1	0.8 ± 0.1	0.6 ± 0.1	0.5 ± 0.1	0.7 ± 0.1	0.6 ± 0.1	< 0.001
**Regional Recurrence SD**	0.2 ± 0.0	0.2 ± 0.0	0.2 ± 0.1	0.2 ± 0.0	0.2 ± 0.0	0.2 ± 0.0	0.2 ± 0.0	0.53
**LIA occ number (cutoff 0) (N)**	72.6 ± 28.6	24.2 ± 12.7	8.6 ± 7.0	45.6 ± 22.4	59.6 ± 32.2	16.2 ± 8.1	37.8 ± 30.7	< 0.001
**LRA occ number (cutoff 0) (N)**	11.8 ± 7.0	3.5 ± 2.1	1.2 ± 2.1	5.5 ± 4.3	10.1 ± 7.9	5.0 ± 5.0	6.2 ± 6.2	0.002
**Focal occ number (cutoff 0) (N)**	27.8 ± 12.3	12.0 ± 10.2	2.6 ± 3.5	17.2 ± 10.5	26.6 ± 15.2	10.8 ± 6.9	16.2 ± 13.4	< 0.001
**LIA perc time (cutoff 0) (N)**	20.5 ± 8.2	6.7 ± 3.6	2.5 ± 2.1	13.0 ± 6.7	16.8 ± 9.3	4.3 ± 2.0	10.6 ± 8.8	< 0.001
**LRA perc time (cutoff 0) (N)**	7.1 ± 5.1	1.7 ± 1.1	0.5 ± 0.8	3.0 ± 2.5	5.7 ± 5.1	2.9 ± 2.8	3.5 ± 3.9	0.003

**Focal perc time (cutoff 0) (N)**	3.4 ± 1.5	1.5 ± 1.3	0.3 ± 0.4	2.1 ± 1.3	3.3 ± 1.8	1.4 ± 0.9	2.0 ± 1.6	< 0.001
**LIA occ number (cutoff 5) (N)**	70.0 ± 28.4	20.0 ± 13.6	4.2 ± 4.8	43.9 ± 21.9	55.6 ± 33.0	10.8 ± 6.0	34.1 ± 31.4	< 0.001
**LRA occ number (cutoff 5) (N)**	10.5 ± 7.4	2.6 ± 2.2	0.9 ± 1.6	4.1 ± 3.8	9.4 ± 7.9	4.6 ± 4.4	5.4 ± 6.0	0.003
**Focal occ number (cutoff 5) (N)**	25.2 ± 12.9	10.2 ± 9.2	1.5 ± 3.1	15.0 ± 11.6	25.1 ± 16.0	9.6 ± 7.1	14.5 ± 13.4	< 0.001
**LIA perc time (cutoff 5) (N)**	19.9 ± 8.2	5.6 ± 3.8	1.3 ± 1.5	12.6 ± 6.6	15.8 ± 9.5	2.9 ± 1.7	9.7 ± 9.0	< 0.001
**LRA perc time (cutoff 5) (N)**	6.6 ± 5.4	1.4 ± 1.1	0.3 ± 0.6	2.5 ± 2.3	5.3 ± 5.0	2.7 ± 2.6	3.1 ± 3.8	0.004
**Focal perc time (cutoff 5) (N)**	3.1 ± 1.6	1.3 ± 1.2	0.2 ± 0.4	1.9 ± 1.4	3.1 ± 2.0	1.2 ± 0.9	1.8 ± 1.6	< 0.001
**LIA occ number (cutoff 30) (N)**	49.0 ± 29.9	8.0 ± 7.7	0.0 ± 0.0	28.2 ± 18.3	38.4 ± 31.4	2.2 ± 2.9	21.0 ± 26.4	< 0.001
**LRA occ number (cutoff 30) (N)**	7.4 ± 5.1	1.2 ± 2.1	0.0 ± 0.0	2.9 ± 4.2	6.0 ± 7.3	2.9 ± 4.1	3.4 ± 4.9	0.015
**Focal occ number (cutoff 30) (N)**	16.9 ± 11.3	5.5 ± 6.2	0.1 ± 0.4	11.8 ± 9.6	21.0 ± 14.0	6.1 ± 4.9	10.2 ± 11.1	< 0.001
**LIA perc time (cutoff 30) (N)**	14.7 ± 9.4	2.2 ± 2.0	0.0 ± 0.0	8.7 ± 5.9	11.4 ± 9.1	0.6 ± 0.8	6.3 ± 8.0	< 0.001

**LRA perc time (cutoff 30) (N)**	4.9 ± 3.9	0.6 ± 1.0	0.0 ± 0.0	1.7 ± 2.5	3.6 ± 4.7	1.7 ± 2.2	2.1 ± 3.2	0.013
**Focal perc time (cutoff 30) (N)**	2.1 ± 1.4	0.7 ± 0.8	0.0 ± 0.0	1.5 ± 1.2	2.6 ± 1.7	0.8 ± 0.6	1.3 ± 1.4	< 0.001

AFCL: atrial fibrillation cycle length; FCA: focal centrifugal activation; LIA: localized irregular activation; LRA: localized rotational activation; PVI: pulmonary vein isolation; SD: standard deviation.

Supplementary Table S2.

Charge density map data by procedural step and region

Region		Baseline (N=66)	Post PVI (N=66)	Post PWI (N=66)	Total (N=198)	p value
**Anterior**	**Global AFCL mean (ms)**	184.4 ± 15.0	198.0 ± 18.0	201.6 ± 17.8	194.0 ± 18.0	0.07
	**Global AFCL SD (ms)**	6.5 ± 1.9	9.6 ± 4.4	7.9 ± 4.2	8.0 ± 3.8	0.16
	**Global AFCL CovC**	3.6 ± 1.1	4.8 ± 2.0	3.9 ± 2.1	4.1 ± 1.8	0.28
	**Regional AFCL mean (ms)**	185.4 ± 15.5	197.0 ± 19.5	200.1 ± 20.8	193.6 ± 19.0	0.19
	**Regional AFCL SD (ms)**	6.6 ± 2.6	6.8 ± 2.9	6.7 ± 4.0	6.7 ± 3.0	0.99
	**Regional AFCL CovC**	3.5 ± 1.3	3.4 ± 1.5	3.4 ± 2.2	3.5 ± 1.6	0.97
	**Global Intra Chamber Coherence mean**	0.4 ± 0.1	0.4 ± 0.1	0.4 ± 0.1	0.4 ± 0.1	0.76
	**Global Intra Chamber Coherence max**	0.5 ± 0.1	0.5 ± 0.1	0.5 ± 0.1	0.5 ± 0.1	0.89
	**Global Intra Chamber Coherence SD**	0.1 ± 0.0	0.1 ± 0.0	0.1 ± 0.0	0.1 ± 0.0	0.87
	**Regional Intra Chamber Coherence mean**	0.3 ± 0.1	0.4 ± 0.2	0.4 ± 0.2	0.4 ± 0.2	0.63
	**Regional Intra Chamber Coherence max**	0.5 ± 0.1	0.5 ± 0.2	0.5 ± 0.2	0.5 ± 0.1	0.85

**Regional Intra Chamber Coherence SD**	0.1 ± 0.0	0.1 ± 0.0	0.1 ± 0.0	0.1 ± 0.0	0.92
**Global Recurrence Mean**	0.4 ± 0.1	0.4 ± 0.1	0.4 ± 0.1	0.4 ± 0.1	0.78
**Global Recurrence SD**	0.2 ± 0.0	0.2 ± 0.0	0.2 ± 0.0	0.2 ± 0.0	0.99
**Regional Recurrence Mean**	0.5 ± 0.1	0.6 ± 0.2	0.6 ± 0.1	0.5 ± 0.1	0.46
**Regional Recurrence SD**	0.2 ± 0.0	0.2 ± 0.0	0.2 ± 0.0	0.2 ± 0.0	0.54
**LIA occ number (cutoff 0) (N)**	61.9 ± 31.8	76.6 ± 44.4	72.6 ± 28.6	70.2 ± 35.6	0.63
**LRA occ number (cutoff 0) (N)**	16.0 ± 12.6	17.3 ± 17.4	11.8 ± 7.0	15.3 ± 13.3	0.67
**Focal occ number (cutoff 0) (N)**	29.0 ± 13.3	25.6 ± 13.0	27.8 ± 12.3	27.4 ± 12.6	0.83
**LIA perc time (cutoff 0) (N)**	17.9 ± 10.8	21.8 ± 14.0	20.5 ± 8.2	20.0 ± 11.3	0.73
**LRA perc time (cutoff 0) (N)**	9.3 ± 8.1	9.7 ± 9.6	7.1 ± 5.1	8.9 ± 7.9	0.76
**Focal perc time (cutoff 0) (N)**	3.6 ± 1.7	3.1 ± 1.6	3.4 ± 1.5	3.4 ± 1.6	0.80
**LIA occ number (cutoff 5) (N)**	56.5 ± 29.4	71.7 ± 43.9	70.0 ± 28.4	65.7 ± 34.8	0.56
**LRA occ number (cutoff 5) (N)**	13.5 ± 11.6	16.3 ± 16.8	10.5 ± 7.4	13.7 ± 12.7	0.64
**Focal occ number (cutoff 5) (N)**	26.2 ± 12.8	22.7 ± 13.7	25.2 ± 12.9	24.7 ± 12.8	0.82

	**LIA perc time (cutoff 5) (N)**	16.7 ± 10.1	20.8 ± 14.0	19.9 ± 8.2	19.1 ± 11.0	0.67
	**LRA perc time (cutoff 5) (N)**	8.1 ± 7.6	9.4 ± 9.4	6.6 ± 5.4	8.2 ± 7.7	0.75
	**Focal perc time (cutoff 5) (N)**	3.2 ± 1.6	2.8 ± 1.7	3.1 ± 1.6	3.0 ± 1.6	0.80
	**LIA occ number (cutoff 30) (N)**	31.0 ± 30.0	51.3 ± 38.6	49.0 ± 29.9	43.2 ± 33.6	0.32
	**LRA occ number (cutoff 30) (N)**	10.4 ± 10.5	11.3 ± 14.4	7.4 ± 5.1	9.9 ± 10.9	0.74
	**Focal occ number (cutoff 30) (N)**	20.6 ± 12.3	15.7 ± 14.5	16.9 ± 11.3	17.8 ± 12.7	0.66
	**LIA perc time (cutoff 30) (N)**	9.8 ± 10.2	15.7 ± 12.9	14.7 ± 9.4	13.3 ± 11.1	0.43
	**LRA perc time (cutoff 30) (N)**	6.4 ± 7.2	6.7 ± 8.4	4.9 ± 3.9	6.1 ± 6.8	0.86
	**Focal perc time (cutoff 30) (N)**	2.6 ± 1.5	1.9 ± 1.8	2.1 ± 1.4	2.2 ± 1.6	0.65
**Septal**	**Global AFCL mean (ms)**	184.4 ± 15.0	198.0 ± 18.0	201.6 ± 17.8	194.0 ± 18.0	0.07
	**Global AFCL SD (ms)**	6.5 ± 1.9	9.6 ± 4.4	7.9 ± 4.2	8.0 ± 3.8	0.16
	**Global AFCL CovC**	3.6 ± 1.1	4.8 ± 2.0	3.9 ± 2.1	4.1 ± 1.8	0.28
	**Regional AFCL mean (ms)**	184.9 ± 16.9	196.7 ± 18.0	200.8 ± 19.3	193.5 ± 18.6	0.14
	**Regional AFCL SD (ms)**	4.7 ± 2.2	5.0 ± 2.3	5.2 ± 3.7	4.9 ± 2.6	0.91

**Regional AFCL CovC**	2.5 ± 1.3	2.5 ± 1.0	2.6 ± 1.9	2.6 ± 1.3	0.99
**Global Intra Chamber Coherence mean**	0.4 ± 0.1	0.4 ± 0.1	0.4 ± 0.1	0.4 ± 0.1	0.76
**Global Intra Chamber Coherence max**	0.5 ± 0.1	0.5 ± 0.1	0.5 ± 0.1	0.5 ± 0.1	0.89
**Global Intra Chamber Coherence SD**	0.1 ± 0.0	0.1 ± 0.0	0.1 ± 0.0	0.1 ± 0.0	0.87
**Regional Intra Chamber Coherence mean**	0.4 ± 0.1	0.4 ± 0.2	0.4 ± 0.2	0.4 ± 0.1	0.64
**Regional Intra Chamber Coherence max**	0.5 ± 0.1	0.5 ± 0.2	0.5 ± 0.1	0.5 ± 0.1	0.92
**Regional Intra Chamber Coherence SD**	0.1 ± 0.0	0.0 ± 0.0	0.1 ± 0.0	0.1 ± 0.0	0.56
**Global Recurrence Mean**	0.4 ± 0.1	0.4 ± 0.1	0.4 ± 0.1	0.4 ± 0.1	0.78
**Global Recurrence SD**	0.2 ± 0.0	0.2 ± 0.0	0.2 ± 0.0	0.2 ± 0.0	0.99
**Regional Recurrence Mean**	0.6 ± 0.1	0.7 ± 0.1	0.7 ± 0.1	0.6 ± 0.1	0.46
**Regional Recurrence SD**	0.2 ± 0.0	0.2 ± 0.0	0.2 ± 0.0	0.2 ± 0.0	0.42
**LIA occ number (cutoff 0) (N)**	28.6 ± 17.5	35.0 ± 27.1	24.2 ± 12.7	29.8 ± 20.4	0.53
**LRA occ number (cutoff 0) (N)**	6.9 ± 9.1	6.4 ± 6.3	3.5 ± 2.1	5.8 ± 6.8	0.54
**Focal occ number (cutoff 0) (N)**	9.6 ± 7.7	11.3 ± 8.7	12.0 ± 10.2	10.9 ± 8.5	0.83

**LIA perc time (cutoff 0) (N)**	8.1 ± 5.6	9.1 ± 7.3	6.7 ± 3.6	8.1 ± 5.8	0.69
**LRA perc time (cutoff 0) (N)**	3.8 ± 5.4	3.4 ± 3.3	1.7 ± 1.1	3.1 ± 3.8	0.49
**Focal perc time (cutoff 0) (N)**	1.2 ± 0.9	1.4 ± 1.0	1.5 ± 1.3	1.3 ± 1.0	0.77
**LIA occ number (cutoff 5) (N)**	24.5 ± 17.3	26.6 ± 21.8	20.0 ± 13.6	24.1 ± 17.9	0.74
**LRA occ number (cutoff 5) (N)**	5.8 ± 8.7	4.8 ± 5.4	2.6 ± 2.2	4.6 ± 6.2	0.55
**Focal occ number (cutoff 5) (N)**	8.0 ± 7.5	9.7 ± 8.1	10.2 ± 9.2	9.2 ± 7.9	0.81
**LIA perc time (cutoff 5) (N)**	7.1 ± 5.5	7.1 ± 6.0	5.6 ± 3.8	6.7 ± 5.1	0.78
**LRA perc time (cutoff 5) (N)**	3.3 ± 5.3	2.6 ± 2.8	1.4 ± 1.1	2.5 ± 3.6	0.53
**Focal perc time (cutoff 5) (N)**	1.0 ± 0.9	1.2 ± 1.0	1.3 ± 1.2	1.1 ± 1.0	0.75
**LIA occ number (cutoff 30) (N)**	6.7 ± 9.8	10.4 ± 11.3	8.0 ± 7.7	8.4 ± 9.7	0.69
**LRA occ number (cutoff 30) (N)**	3.1 ± 6.2	2.3 ± 3.2	1.2 ± 2.1	2.3 ± 4.3	0.67
**Focal occ number (cutoff 30) (N)**	4.6 ± 5.3	6.5 ± 8.5	5.5 ± 6.2	5.6 ± 6.7	0.81
**LIA perc time (cutoff 30) (N)**	2.0 ± 3.1	2.8 ± 3.1	2.2 ± 2.0	2.4 ± 2.8	0.79
**LRA perc time (cutoff 30) (N)**	1.9 ± 3.9	1.2 ± 1.8	0.6 ± 1.0	1.3 ± 2.6	0.59

	**Focal perc time (cutoff 30) (N)**	0.6 ± 0.7	0.8 ± 1.0	0.7 ± 0.8	0.7 ± 0.8	0.83
**Lateral**	**Global AFCL mean (ms)**	184.4 ± 15.0	198.0 ± 18.0	201.6 ± 17.8	194.0 ± 18.0	0.07
	**Global AFCL SD (ms)**	6.5 ± 1.9	9.6 ± 4.4	7.9 ± 4.2	8.0 ± 3.8	0.16
	**Global AFCL CovC**	3.6 ± 1.1	4.8 ± 2.0	3.9 ± 2.1	4.1 ± 1.8	0.28
	**Regional AFCL mean (ms)**	188.1 ± 14.6	204.7 ± 21.7	208.9 ± 20.1	199.7 ± 20.4	0.048
	**Regional AFCL SD (ms)**	4.7 ± 1.8	7.2 ± 4.2	6.0 ± 3.7	6.0 ± 3.4	0.24
	**Regional AFCL CovC**	2.5 ± 1.0	3.5 ± 2.0	2.8 ± 1.7	3.0 ± 1.6	0.38
	**Global Intra Chamber Coherence mean**	0.4 ± 0.1	0.4 ± 0.1	0.4 ± 0.1	0.4 ± 0.1	0.76
	**Global Intra Chamber Coherence max**	0.5 ± 0.1	0.5 ± 0.1	0.5 ± 0.1	0.5 ± 0.1	0.89
	**Global Intra Chamber Coherence SD**	0.1 ± 0.0	0.1 ± 0.0	0.1 ± 0.0	0.1 ± 0.0	0.87
	**Regional Intra Chamber Coherence mean**	0.4 ± 0.1	0.4 ± 0.1	0.5 ± 0.1	0.4 ± 0.1	0.50
	**Regional Intra Chamber Coherence max**	0.5 ± 0.1	0.5 ± 0.2	0.5 ± 0.1	0.5 ± 0.1	0.76
	**Regional Intra Chamber Coherence SD**	0.0 ± 0.0	0.0 ± 0.0	0.0 ± 0.0	0.0 ± 0.0	0.66
	**Global Recurrence Mean**	0.4 ± 0.1	0.4 ± 0.1	0.4 ± 0.1	0.4 ± 0.1	0.78

**Global Recurrence SD**	0.2 ± 0.0	0.2 ± 0.0	0.2 ± 0.0	0.2 ± 0.0	0.99
**Regional Recurrence Mean**	0.7 ± 0.1	0.8 ± 0.1	0.8 ± 0.1	0.8 ± 0.1	0.56
**Regional Recurrence SD**	0.2 ± 0.0	0.2 ± 0.0	0.2 ± 0.1	0.2 ± 0.0	0.35
**LIA occ number (cutoff 0) (N)**	17.1 ± 15.4	17.4 ± 14.0	8.6 ± 7.0	14.9 ± 13.3	0.30
**LRA occ number (cutoff 0) (N)**	2.4 ± 2.6	3.2 ± 5.4	1.2 ± 2.1	2.4 ± 3.7	0.55
**Focal occ number (cutoff 0) (N)**	7.3 ± 3.1	8.1 ± 5.7	2.6 ± 3.5	6.3 ± 4.8	0.027
**LIA perc time (cutoff 0) (N)**	4.6 ± 4.2	4.7 ± 4.1	2.5 ± 2.1	4.1 ± 3.7	0.37
**LRA perc time (cutoff 0) (N)**	1.1 ± 1.4	1.6 ± 2.5	0.5 ± 0.8	1.1 ± 1.8	0.43
**Focal perc time (cutoff 0) (N)**	0.9 ± 0.4	0.9 ± 0.6	0.3 ± 0.4	0.7 ± 0.6	0.034
**LIA occ number (cutoff 5) (N)**	11.5 ± 12.4	11.7 ± 9.0	4.2 ± 4.8	9.6 ± 9.9	0.20
**LRA occ number (cutoff 5) (N)**	1.8 ± 2.1	2.5 ± 4.8	0.9 ± 1.6	1.8 ± 3.3	0.56
**Focal occ number (cutoff 5) (N)**	5.3 ± 3.0	6.7 ± 5.8	1.5 ± 3.1	4.8 ± 4.6	0.043
**LIA perc time (cutoff 5) (N)**	3.2 ± 3.5	3.3 ± 2.7	1.3 ± 1.5	2.7 ± 2.9	0.26
**LRA perc time (cutoff 5) (N)**	0.9 ± 1.1	1.2 ± 2.1	0.3 ± 0.6	0.8 ± 1.5	0.51

	**Focal perc time (cutoff 5) (N)**	0.6 ± 0.4	0.8 ± 0.7	0.2 ± 0.4	0.6 ± 0.6	0.06
	**LIA occ number (cutoff 30) (N)**	4.7 ± 9.1	3.0 ± 5.2	0.0 ± 0.0	2.8 ± 6.4	0.29
	**LRA occ number (cutoff 30) (N)**	0.3 ± 0.9	1.4 ± 4.2	0.0 ± 0.0	0.6 ± 2.6	0.47
	**Focal occ number (cutoff 30) (N)**	1.9 ± 2.5	3.6 ± 4.5	0.1 ± 0.4	2.1 ± 3.4	0.07
	**LIA perc time (cutoff 30) (N)**	1.3 ± 2.6	0.8 ± 1.4	0.0 ± 0.0	0.8 ± 1.8	0.29
	**LRA perc time (cutoff 30) (N)**	0.1 ± 0.4	0.6 ± 1.8	0.0 ± 0.0	0.3 ± 1.1	0.43
	**Focal perc time (cutoff 30) (N)**	0.2 ± 0.3	0.5 ± 0.6	0.0 ± 0.0	0.3 ± 0.4	0.07
**Roof**	**Global AFCL mean (ms)**	184.4 ± 15.0	198.0 ± 18.0	201.6 ± 17.8	194.0 ± 18.0	0.07
	**Global AFCL SD (ms)**	6.5 ± 1.9	9.6 ± 4.4	7.9 ± 4.2	8.0 ± 3.8	0.16
	**Global AFCL CovC**	3.6 ± 1.1	4.8 ± 2.0	3.9 ± 2.1	4.1 ± 1.8	0.28
	**Regional AFCL mean (ms)**	180.1 ± 14.8	194.8 ± 20.2	199.0 ± 15.7	190.5 ± 18.5	0.05
	**Regional AFCL SD(ms)**	4.0 ± 1.9	4.7 ± 3.3	6.7 ± 4.8	5.0 ± 3.4	0.25
	**Regional AFCL CovC**	2.2 ± 0.9	2.4 ± 1.6	3.3 ± 2.4	2.6 ± 1.7	0.34
	**Global Intra Chamber Coherence mean**	0.4 ± 0.1	0.4 ± 0.1	0.4 ± 0.1	0.4 ± 0.1	0.76

**Global Intra Chamber Coherence max**	0.5 ± 0.1	0.5 ± 0.1	0.5 ± 0.1	0.5 ± 0.1	0.89
**Global Intra Chamber Coherence SD**	0.1 ± 0.0	0.1 ± 0.0	0.1 ± 0.0	0.1 ± 0.0	0.87
**Regional Intra Chamber Coherence mean**	0.4 ± 0.1	0.4 ± 0.2	0.4 ± 0.1	0.4 ± 0.1	0.69
**Regional Intra Chamber Coherence max**	0.5 ± 0.1	0.5 ± 0.2	0.5 ± 0.1	0.5 ± 0.1	0.85
**Regional Intra Chamber Coherence SD**	0.1 ± 0.0	0.0 ± 0.0	0.1 ± 0.0	0.1 ± 0.0	0.46
**Global Recurrence Mean**	0.4 ± 0.1	0.4 ± 0.1	0.4 ± 0.1	0.4 ± 0.1	0.78
**Global Recurrence SD**	0.2 ± 0.0	0.2 ± 0.0	0.2 ± 0.0	0.2 ± 0.0	0.99
**Regional Recurrence Mean**	0.6 ± 0.1	0.7 ± 0.1	0.6 ± 0.1	0.6 ± 0.1	0.06
**Regional Recurrence SD**	0.2 ± 0.0	0.2 ± 0.1	0.2 ± 0.0	0.2 ± 0.0	0.65
**LIA occ number (cutoff 0) (N)**	45.8 ± 32.8	38.4 ± 21.5	45.6 ± 22.4	43.0 ± 25.8	0.76
**LRA occ number (cutoff 0) (N)**	11.0 ± 8.4	7.5 ± 8.7	5.5 ± 4.3	8.3 ± 7.8	0.03
**Focal occ number (cutoff 0) (N)**	17.7 ± 15.6	10.9 ± 7.1	17.2 ± 10.5	15.1 ± 11.8	0.34
**LIA perc time (cutoff 0) (N)**	13.2 ± 9.7	11.2 ± 6.9	13.0 ± 6.7	12.4 ± 7.8	0.81
**LRA perc time (cutoff 0) (N)**	6.0 ± 4.6	4.7 ± 6.6	3.0 ± 2.5	4.7 ± 5.0	0.045

	**Focal perc time (cutoff 0) (N)**	2.2 ± 1.9	1.3 ± 0.9	2.1 ± 1.3	1.9 ± 1.5	0.34
	**LIA occ number (cutoff 5) (N)**	43.8 ± 34.2	34.2 ± 20.4	43.9 ± 21.9	40.3 ± 26.1	0.64
	**LRA occ number (cutoff 5) (N)**	10.1 ± 8.3	6.7 ± 8.0	4.1 ± 3.8	7.3 ± 7.4	0.05
	**Focal occ number (cutoff 5) (N)**	17.3 ± 16.0	9.4 ± 7.0	15.0 ± 11.6	13.8 ± 12.2	0.31
	**LIA perc time (cutoff 5) (N)**	12.7 ± 10.1	10.3 ± 6.9	12.6 ± 6.6	11.8 ± 8.0	0.74
	**LRA perc time (cutoff 5) (N)**	5.5 ± 4.5	4.3 ± 6.3	2.5 ± 2.3	4.2 ± 4.8	0.07
	**Focal perc time (cutoff 5) (N)**	2.1 ± 2.0	1.2 ± 0.8	1.9 ± 1.4	1.7 ± 1.5	0.30
	**LIA occ number (cutoff 30) (N)**	28.5 ± 27.7	22.5 ± 22.1	28.2 ± 18.3	26.2 ± 22.8	0.80
	**LRA occ number (cutoff 30) (N)**	6.5 ± 6.6	4.0 ± 7.8	2.9 ± 4.2	4.6 ± 6.5	0.46
	**Focal occ number (cutoff 30) (N)**	14.2 ± 15.2	6.2 ± 6.5	11.8 ± 9.6	10.6 ± 11.4	0.25
	**LIA perc time (cutoff 30) (N)**	8.7 ± 8.7	6.9 ± 7.5	8.7 ± 5.9	8.1 ± 7.4	0.83
	**LRA perc time (cutoff 30) (N)**	3.6 ± 3.7	2.8 ± 6.4	1.7 ± 2.5	2.8 ± 4.6	0.69
	**Focal perc time (cutoff 30) (N)**	1.8 ± 1.9	0.8 ± 0.8	1.5 ± 1.2	1.3 ± 1.4	0.23
**Posterior**	**Global AFCL mean (ms)**	184.4 ± 15.0	198.0 ± 18.0	201.6 ± 17.8	194.0 ± 18.0	0.07

**Global AFCL SD (ms)**	6.5 ± 1.9	9.6 ± 4.4	7.9 ± 4.2	8.0 ± 3.8	0.16
**Global AFCL CovC**	3.6 ± 1.1	4.8 ± 2.0	3.9 ± 2.1	4.1 ± 1.8	0.28
**Regional AFCL mean (ms)**	182.1 ± 14.5	196.8 ± 18.2	200.0 ± 15.3	192.3 ± 17.5	0.04
**Regional AFCL SD (ms)**	5.3 ± 2.5	7.6 ± 4.1	6.1 ± 2.9	6.4 ± 3.3	0.25
**Regional AFCL CovC**	2.9 ± 1.5	3.8 ± 1.9	3.1 ± 1.6	3.3 ± 1.7	0.45
**Global Intra Chamber Coherence mean**	0.4 ± 0.1	0.4 ± 0.1	0.4 ± 0.1	0.4 ± 0.1	0.76
**Global Intra Chamber Coherence max**	0.5 ± 0.1	0.5 ± 0.1	0.5 ± 0.1	0.5 ± 0.1	0.89
**Global Intra Chamber Coherence SD**	0.1 ± 0.0	0.1 ± 0.0	0.1 ± 0.0	0.1 ± 0.0	0.87
**Regional Intra Chamber Coherence mean**	0.4 ± 0.1	0.4 ± 0.2	0.4 ± 0.2	0.4 ± 0.1	0.92
**Regional Intra Chamber Coherence max**	0.5 ± 0.1	0.5 ± 0.1	0.5 ± 0.1	0.5 ± 0.1	0.92
**Regional Intra Chamber Coherence SD**	0.1 ± 0.0	0.1 ± 0.0	0.1 ± 0.0	0.1 ± 0.0	0.83
**Global Recurrence Mean**	0.4 ± 0.1	0.4 ± 0.1	0.4 ± 0.1	0.4 ± 0.1	0.78
**Global Recurrence SD**	0.2 ± 0.0	0.2 ± 0.0	0.2 ± 0.0	0.2 ± 0.0	0.99
**Regional Recurrence Mean**	0.5 ± 0.1	0.6 ± 0.1	0.5 ± 0.1	0.5 ± 0.1	0.92

**Regional Recurrence SD**	0.2 ± 0.0	0.2 ± 0.0	0.2 ± 0.0	0.2 ± 0.0	0.69
**LIA occ number (cutoff 0) (N)**	74.7 ± 33.2	65.2 ± 40.3	59.6 ± 32.2	67.2 ± 35.1	0.65
**LRA occ number (cutoff 0) (N)**	16.9 ± 9.8	12.6 ± 14.5	10.1 ± 7.9	13.5 ± 11.4	0.042
**Focal occ number (cutoff 0) (N)**	23.3 ± 8.7	26.1 ± 11.8	26.6 ± 15.2	25.2 ± 11.5	0.79
**LIA perc time (cutoff 0) (N)**	21.7 ± 10.8	18.2 ± 12.0	16.8 ± 9.3	19.1 ± 10.7	0.59
**LRA perc time (cutoff 0) (N)**	8.7 ± 5.5	6.4 ± 6.7	5.7 ± 5.1	7.1 ± 5.8	0.048
**Focal perc time (cutoff 0) (N)**	2.9 ± 1.1	3.2 ± 1.4	3.3 ± 1.8	3.1 ± 1.4	0.79
**LIA occ number (cutoff 5) (N)**	71.5 ± 34.1	60.4 ± 38.6	55.6 ± 33.0	63.2 ± 35.0	0.60
**LRA occ number (cutoff 5) (N)**	15.2 ± 8.7	11.4 ± 13.5	9.4 ± 7.9	12.2 ± 10.5	0.08
**Focal occ number (cutoff 5) (N)**	21.6 ± 9.1	23.5 ± 12.9	25.1 ± 16.0	23.3 ± 12.2	0.83
**LIA perc time (cutoff 5) (N)**	20.9 ± 11.0	17.1 ± 11.8	15.8 ± 9.5	18.2 ± 10.8	0.57
**LRA perc time (cutoff 5) (N)**	8.2 ± 5.2	5.9 ± 6.3	5.3 ± 5.0	6.5 ± 5.6	0.09
**Focal perc time (cutoff 5) (N)**	2.7 ± 1.1	2.9 ± 1.5	3.1 ± 2.0	2.9 ± 1.5	0.84
**LIA occ number (cutoff 30) (N)**	54.5 ± 28.5	40.2 ± 30.0	38.4 ± 31.4	45.0 ± 29.7	0.42

	**LRA occ number (cutoff 30) (N)**	10.5 ± 6.8	7.8 ± 8.9	6.0 ± 7.3	8.3 ± 7.7	0.16
	**Focal occ number (cutoff 30) (N)**	15.6 ± 10.2	16.3 ± 12.7	21.0 ± 14.0	17.3 ± 12.0	0.61
	**LIA perc time (cutoff 30) (N)**	16.6 ± 9.5	12.1 ± 9.9	11.4 ± 9.1	13.6 ± 9.5	0.42
	**LRA perc time (cutoff 30) (N)**	5.7 ± 4.1	4.5 ± 4.8	3.6 ± 4.7	4.7 ± 4.5	0.11
	**Focal perc time (cutoff 30) (N)**	1.9 ± 1.3	2.0 ± 1.5	2.6 ± 1.7	2.1 ± 1.5	0.59
**Inferior**	**Global AFCL mean (ms)**	184.4 ± 15.0	198.0 ± 18.0	201.6 ± 17.8	194.0 ± 18.0	0.07
	**Global AFCL SD (ms)**	6.5 ± 1.9	9.6 ± 4.4	7.9 ± 4.2	8.0 ± 3.8	0.16
	**Global AFCL CovC**	3.6 ± 1.1	4.8 ± 2.0	3.9 ± 2.1	4.1 ± 1.8	0.28
	**Regional AFCL mean (ms)**	186.4 ± 14.6	198.0 ± 17.2	202.0 ± 18.8	194.8 ± 17.5	0.12
	**Regional AFCL SD (ms)**	5.4 ± 2.3	5.8 ± 2.3	5.9 ± 3.9	5.7 ± 2.7	0.93
	**Regional AFCL CovC**	3.0 ± 1.4	2.9 ± 1.0	2.9 ± 1.7	2.9 ± 1.3	0.98
	**Global Intra Chamber Coherence mean**	0.4 ± 0.1	0.4 ± 0.1	0.4 ± 0.1	0.4 ± 0.1	0.76
	**Global Intra Chamber Coherence max**	0.5 ± 0.1	0.5 ± 0.1	0.5 ± 0.1	0.5 ± 0.1	0.89
	**Global Intra Chamber Coherence SD**	0.1 ± 0.0	0.1 ± 0.0	0.1 ± 0.0	0.1 ± 0.0	0.87

**Regional Intra Chamber Coherence mean**	0.4 ± 0.1	0.4 ± 0.1	0.4 ± 0.1	0.4 ± 0.1	0.72
**Regional Intra Chamber Coherence max**	0.5 ± 0.1	0.5 ± 0.2	0.5 ± 0.1	0.5 ± 0.1	0.84
**Regional Intra Chamber Coherence SD**	0.0 ± 0.0	0.0 ± 0.0	0.0 ± 0.0	0.0 ± 0.0	0.76
**Global Recurrence Mean**	0.4 ± 0.1	0.4 ± 0.1	0.4 ± 0.1	0.4 ± 0.1	0.78
**Global Recurrence SD**	0.2 ± 0.0	0.2 ± 0.0	0.2 ± 0.0	0.2 ± 0.0	0.99
**Regional Recurrence Mean**	0.7 ± 0.1	0.7 ± 0.1	0.7 ± 0.1	0.7 ± 0.1	0.19
**Regional Recurrence SD**	0.2 ± 0.0	0.2 ± 0.0	0.2 ± 0.0	0.2 ± 0.0	0.62
**LIA occ number (cutoff 0) (N)**	32.7 ± 22.1	24.7 ± 14.1	16.2 ± 8.1	25.4 ± 17.2	0.011
**LRA occ number (cutoff 0) (N)**	5.9 ± 4.5	5.1 ± 5.5	5.0 ± 5.0	5.4 ± 4.9	0.90
**Focal occ number (cutoff 0) (N)**	10.9 ± 8.2	11.5 ± 6.5	10.8 ± 6.9	11.1 ± 7.0	0.97
**LIA perc time (cutoff 0) (N)**	9.1 ± 6.5	6.7 ± 3.9	4.3 ± 2.0	7.0 ± 5.0	0.013
**LRA perc time (cutoff 0) (N)**	3.2 ± 2.7	2.9 ± 3.2	2.9 ± 2.8	3.0 ± 2.8	0.95
**Focal perc time (cutoff 0) (N)**	1.3 ± 1.0	1.4 ± 0.8	1.4 ± 0.9	1.4 ± 0.9	0.96
**LIA occ number (cutoff 5) (N)**	26.4 ± 22.5	17.5 ± 10.8	10.8 ± 6.0	19.0 ± 16.2	0.019

**LRA occ number (cutoff 5) (N)**	5.3 ± 4.6	4.5 ± 5.6	4.6 ± 4.4	4.8 ± 4.8	0.93
**Focal occ number (cutoff 5) (N)**	9.0 ± 8.2	10.5 ± 6.1	9.6 ± 7.1	9.7 ± 7.0	0.89
**LIA perc time (cutoff 5) (N)**	7.6 ± 6.7	4.8 ± 2.9	2.9 ± 1.7	5.3 ± 4.8	0.09
**LRA perc time (cutoff 5) (N)**	2.9 ± 2.8	2.6 ± 3.2	2.7 ± 2.6	2.7 ± 2.8	0.98
**Focal perc time (cutoff 5) (N)**	1.1 ± 1.0	1.3 ± 0.8	1.2 ± 0.9	1.2 ± 0.9	0.89
**LIA occ number (cutoff 30) (N)**	14.6 ± 18.8	6.5 ± 6.4	2.2 ± 2.9	8.3 ± 12.8	0.09
**LRA occ number (cutoff 30) (N)**	2.4 ± 2.8	3.0 ± 4.5	2.9 ± 4.1	2.7 ± 3.7	0.92
**Focal occ number (cutoff 30) (N)**	3.5 ± 3.9	6.6 ± 5.5	6.1 ± 4.9	5.3 ± 4.8	0.27
**LIA perc time (cutoff 30) (N)**	4.4 ± 5.8	1.8 ± 1.8	0.6 ± 0.8	2.4 ± 3.9	0.08
**LRA perc time (cutoff 30) (N)**	1.4 ± 1.8	1.7 ± 2.6	1.7 ± 2.2	1.6 ± 2.2	0.93
**Focal perc time (cutoff 30) (N)**	0.4 ± 0.5	0.8 ± 0.7	0.8 ± 0.6	0.7 ± 0.6	0.27

AFCL: atrial fibrillation cycle length; FCA: focal centrifugal activation; LIA: localized irregular activation; LRA: localized rotational activation; PVI: pulmonary vein isolation; SD: standard deviation.

SUPPLEMENTARY FIGURES

Supplementary Figure S1. Left atrium regional segmentation

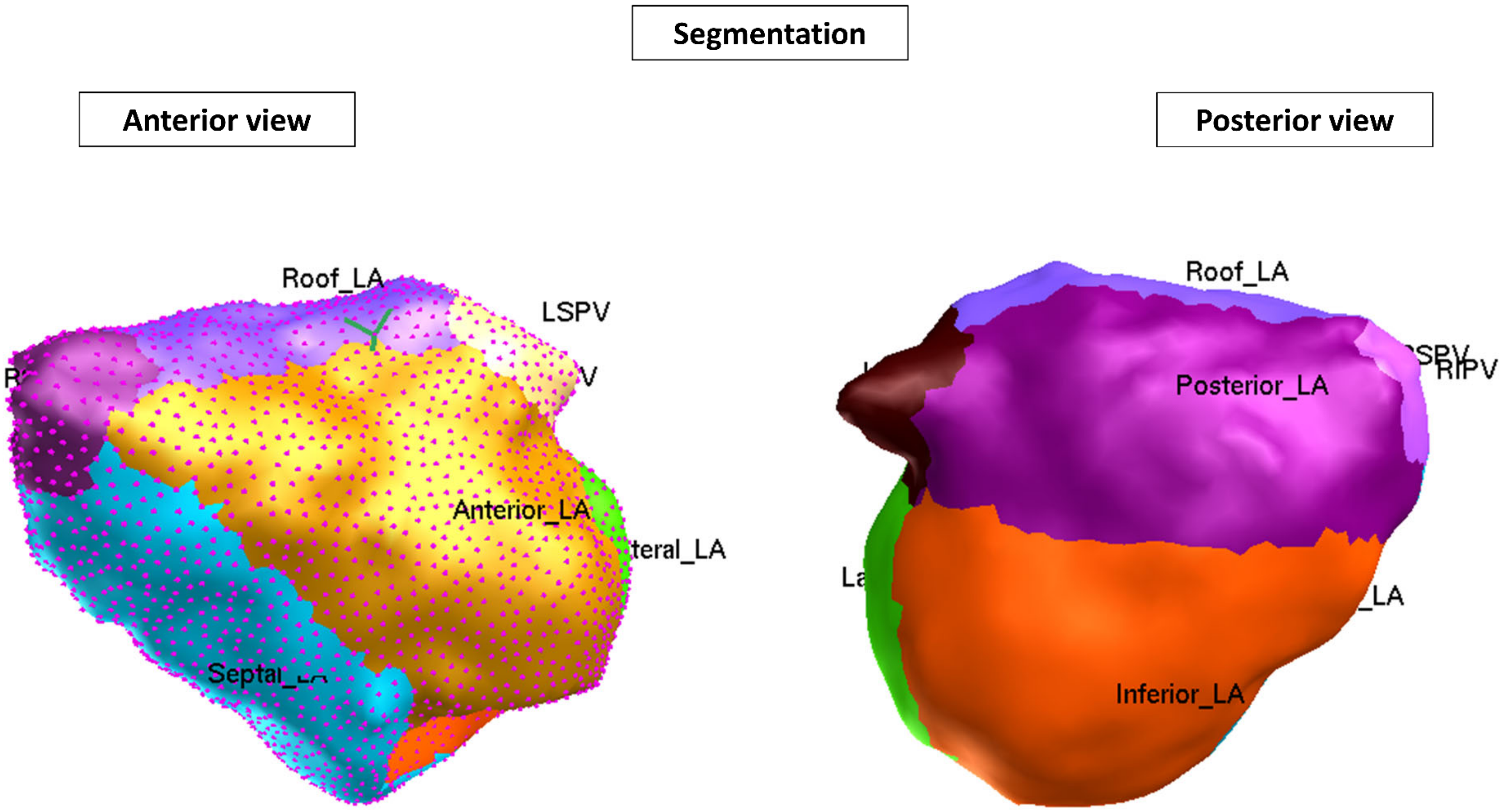

Left atrium regional segmentation. On the left anterior-posterior view, on the right posterior-anterior view. For the current study, LA maps were divided into the following 6 regions: anterior, septal, lateral, roof, posterior, inferior. The analysis was performed accordingly, for global LA map and for each regional AF segment.
